# A multi-technique ensemble model leveraging attention mechanism and image processing for enhanced colorectal tumor detection

**DOI:** 10.1038/s41598-025-25986-2

**Published:** 2025-12-03

**Authors:** B. L. Dharshini, A Arivarasi, B. Prashanth Kumar, J. K. Varsha, S. Pavithraa

**Affiliations:** Vellore Institute of Technology, Chennai, India

**Keywords:** Colorectal tumor detection, Histopathological images, Convolutional neural network (CNN), Transfer learning, Attention mechanisms, Medical image analysis, Watershed algorithm, Distance transform., Medical imaging, Imaging techniques

## Abstract

This research introduces an improved method for identifying colorectal tumors through a combination of deep convolutional neural networks (CNNs), transfer learning, and sophisticated image processing techniques used on histopathological images. The suggested ensemble—based on ResNet50 and enhanced with a dual attention mechanism—surpasses individual model architectures by enhancing both accuracy and interpretability, allowing the model to emphasize crucial tissue areas pertinent to diagnosis. Segmentation techniques, such as watershed and distance transform, are utilized to define tumor margins and possible lesion regions. The dataset, obtained from Kather et al. (2019), includes 5,000 histopathological images spanning eight unique categories (tumor, stroma, complex, lymph, debris, mucosa, adipose, empty). The experimental findings demonstrate impressive results, achieving a training accuracy of 98.74%, a validation accuracy of 94.35%, an F1-score of 0.94, a recall of 0.94, a precision of 0.95, a specificity of 0.96, and a Cohen’s kappa score of 0.9354, signifying outstanding inter-class consensus. These results showcase the model’s strength across different class distributions and emphasize its possible clinical value in aiding the early identification and management of colorectal cancer.

## Introduction

Colorectal cancer remains a major global health challenge, with increasing demand for advanced diagnostic tools^1^ to ensure timely and accurate detection^2–4^. Originating in the colon or rectum, it typically develops from precancerous polyps^5^. According to the latest World Health Organization (WHO) estimates^6^, colorectal cancer is the third most common cancer worldwide, accounting for approximately 1.9 million new cases and 930,000 deaths annually as of 2023^7^. The Indian Council of Medical Research (ICMR) further reports a rising incidence in India, particularly in urban regions, with post-2016 data showing continued growth in age-standardized incidence beyond the earlier rate of 9.3 per 100,000 population^8^. Early detection through screening methods such as colonoscopy, sigmoidoscopy, fecal occult blood test (FOBT), and fecal immunochemical testing (FIT) is critical for reducing mortality^9^. Histopathology plays an equally central role in staging colorectal cancer using the TNM classification system^10^, distinguishing between benign and malignant tumors, guiding treatment, and monitoring therapeutic response^11,12^. However, variability in human interpretation and challenges such as tumor heterogeneity can impact diagnostic consistency.

**Fig. 1:**
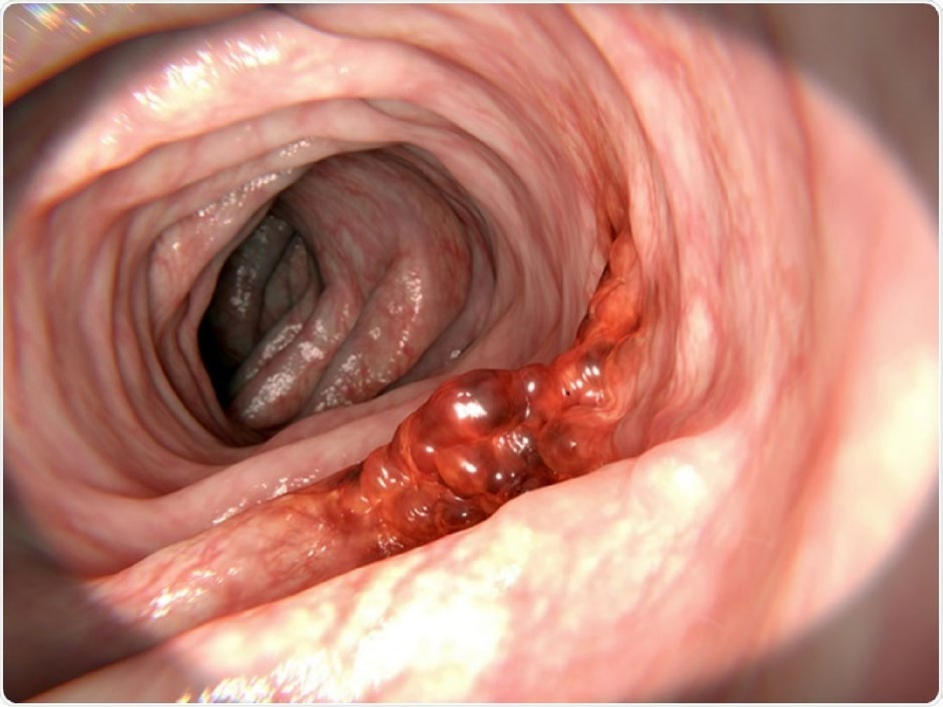
Colorectal cancer 3d rendering. *Image Credit: Juan Gaertner / Shutterstock* (34).

**Fig. 2:**
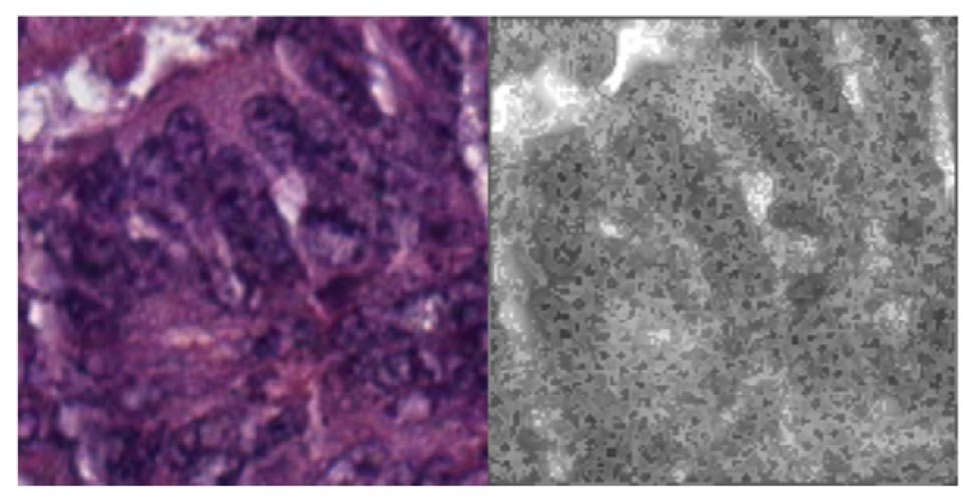
Original Image vs. Marked Image: Features Analysis for Region Identification.

Figure 1 illustrates a general 3D rendering of colorectal cancer for context (illustrative, not study data). Histopathological images provide highly detailed insights into tissue microarchitecture, making them indispensable for computer-aided diagnosis^13^. This research builds on these strengths by integrating Convolutional Neural Networks (CNNs) with advanced image processing techniques^14^. For example, watershed segmentation delineates tissue regions, while distance transform highlights tumor boundaries and edges (Fig. 2)^15^.

By incorporating transfer learning, the model harnesses pre-trained features from various neural network architectures, accelerating convergence and bolstering performance^16,17^. The inclusion of attention mechanisms enhances interpretability, shedding light on the crucial regions within histopathological images^18^ that contribute to accurate tumor identification^19^. This research aims to not only improve the precision of colorectal tumor detection but also to contribute to the broader field of medical image analysis, potentially revolutionizing early diagnosis, and intervention strategies for colorectal cancer patients.

## Related works

Research in colorectal tumor detection has increasingly intersected with medical imaging, deep learning^20^, and pattern recognition, with a clear trend toward ensemble-based deep neural networks (DNNs). Prior works can be organized into several thematic groups.

### CNN-based models

Early studies such as Karthikeyan et al.^21^ applied CNNs with ranking algorithms for colorectal cancer detection, highlighting the importance of deep learning for tumor identification in histopathology. Similarly, Azar et al.^22] and [23^ developed an automated CNN-based system for detection and segmentation, while Zhao et al.^24,25^ combined deep graph convolution with multiple-instance learning (MIL) for predicting lymph node metastasis, demonstrating the power of graph-based feature extraction^26^.

### Ensemble approaches

Li et al.^27] and [28^ proposed multi-CNN fusion, showing improved robustness compared with single architectures. However, most of these approaches faced challenges due to limited dataset size and tumor heterogeneity, which constrained generalizability.

### Transfer learning

Zhang et al.^29^ introduced attention-guided transfer learning for colorectal histopathology, leveraging pre-trained CNNs to capture region-specific features. Reis et al.^16^ expanded this by using general histopathology datasets for cross-domain learning, underscoring the potential of transfer learning to accelerate training and improve performance.

### Attention mechanisms

Attention has emerged as a powerful tool for interpretability. Yamashita et al.^30^ demonstrated spatiotemporal modeling using 3D CNNs, while more recent works like Amin et al.^31,32^ and Shafi et al.^33^ extended attention-based designs to diverse medical tasks (e.g., lung cancer CT scans), improving localization of clinically relevant features.

### Image processing integration

Methods such as watershed segmentation and distance transforms remain underexplored in colorectal tumor detection, though they have proven effective in enhancing boundary detection and region separation in other medical imaging applications (29).

Despite these contributions, several **gaps remain**. Many single-CNN studies^27,29,34^ lack interpretability, others rely on small datasets^30,35^, and few explore multi-technique integration. Recent advances underscore the need for frameworks that combine **ensembles**,** transfer learning**,** attention**,** and image processing** to improve both accuracy and clinical usability^6–38^.

To address these gaps, this research introduces a multi-technique ensemble framework with double attention and preprocessing-based segmentation. Unlike prior works, it directly integrates complementary strategies into a unified model.

## Methodology

**Fig. 3:**
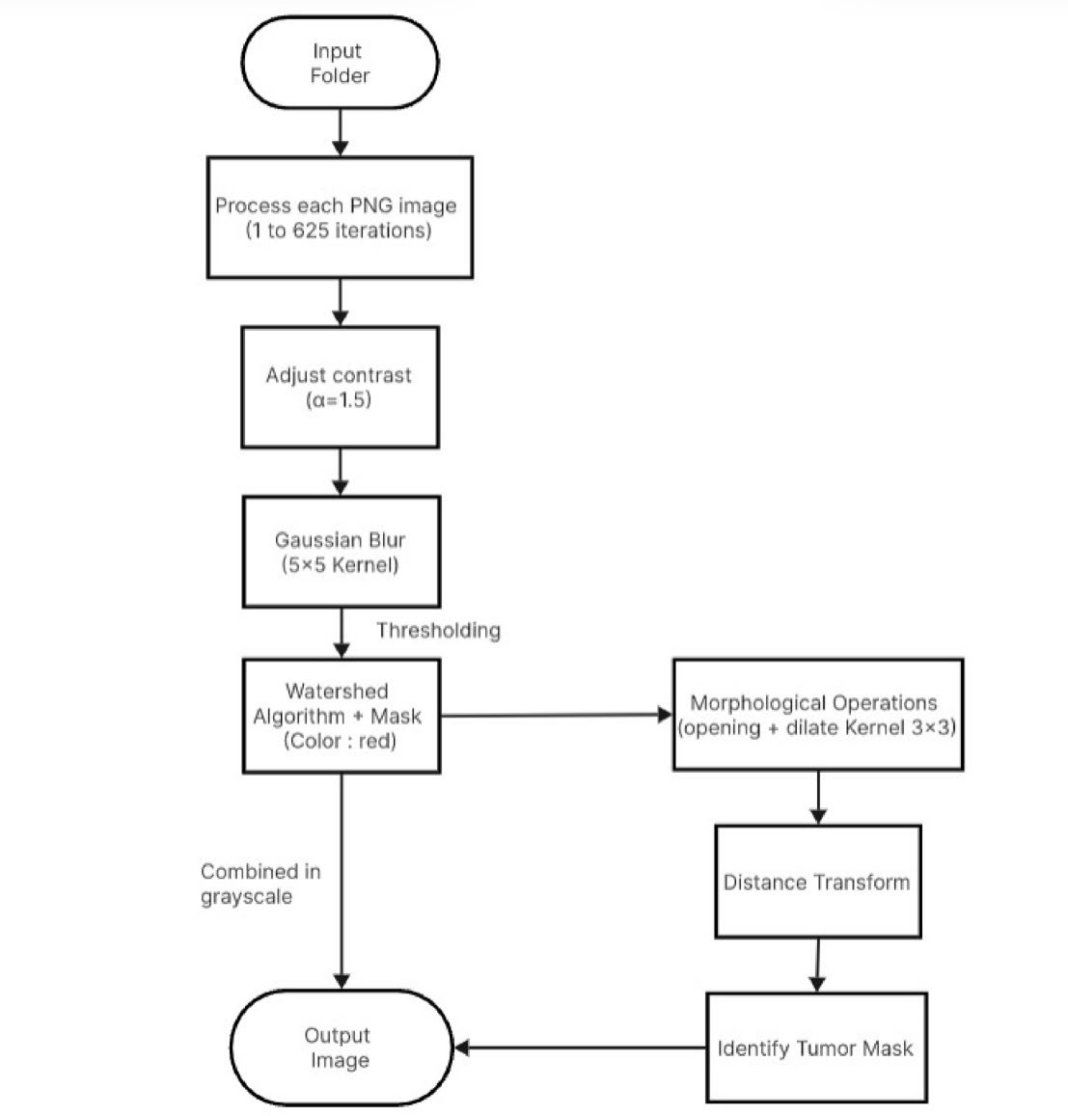
Image processing flow used for the potential tumor identification.

A key paradigm in the field of colorectal tumor detection from histopathological images is the convolutional neural network (CNN). The durability and accuracy of tumor identification are improved by ensemble models, which combine the advantages of several neural network architectures. These models use transfer learning to combine pre-trained features from several sources. By combining CNNs with attention mechanisms, this work pushes this frontier farther while highlighting the significance of pattern recognition and encouraging interpretability. This novel strategy aims to improve the accuracy of colorectal cancer detection while advancing the field’s understanding of the relationship between pattern recognition and medical image analysis in the context of research papers (Fig. 3).

To improve the accuracy and robustness of the model, we used a variety of image processing techniques along with deep learning approaches in this research study on colorectal tumor identification. The flow and relationship between each parameter provide a systematic approach to colon tumor detection.

### Workflow overview

The proposed pipeline (Fig. 3) follows a structured sequence:

1. **Preprocessing and Segmentation**: Images are first enhanced through contrast adjustment, Gaussian blur (kernel size = 5 × 5), and morphological operations (structuring element = 3 × 3) to reduce noise and artifacts. The distance transform emphasizes tumor boundaries, while the watershed algorithm segments tissues into distinct regions.
2. **Data Augmentation**: To increase dataset variability and improve model robustness, augmentation techniques are applied, including rotations (± 30°), zoom (0.2), horizontal/vertical flips, and brightness adjustments.
3. **Feature Extraction and Classification**: Preprocessed images are fed into ResNet50 (selected for its balance of depth and computational efficiency), alongside Inception-ResNetV2 and a custom CNN. Their feature outputs are concatenated within the ensemble fusion layer. Training is optimized with the Adam optimizer (learning rate = 0.001, batch size = 32, epochs = 100).
4. **Attention Integration**:

- *Channel attention* (Eq. 1) applies global average pooling and a sigmoid activation to recalibrate feature map importance.
- *Spatial attention* (Eq. 2) uses 2D convolution over pooled descriptors to highlight spatially significant pixels.

**Fig. 4:**
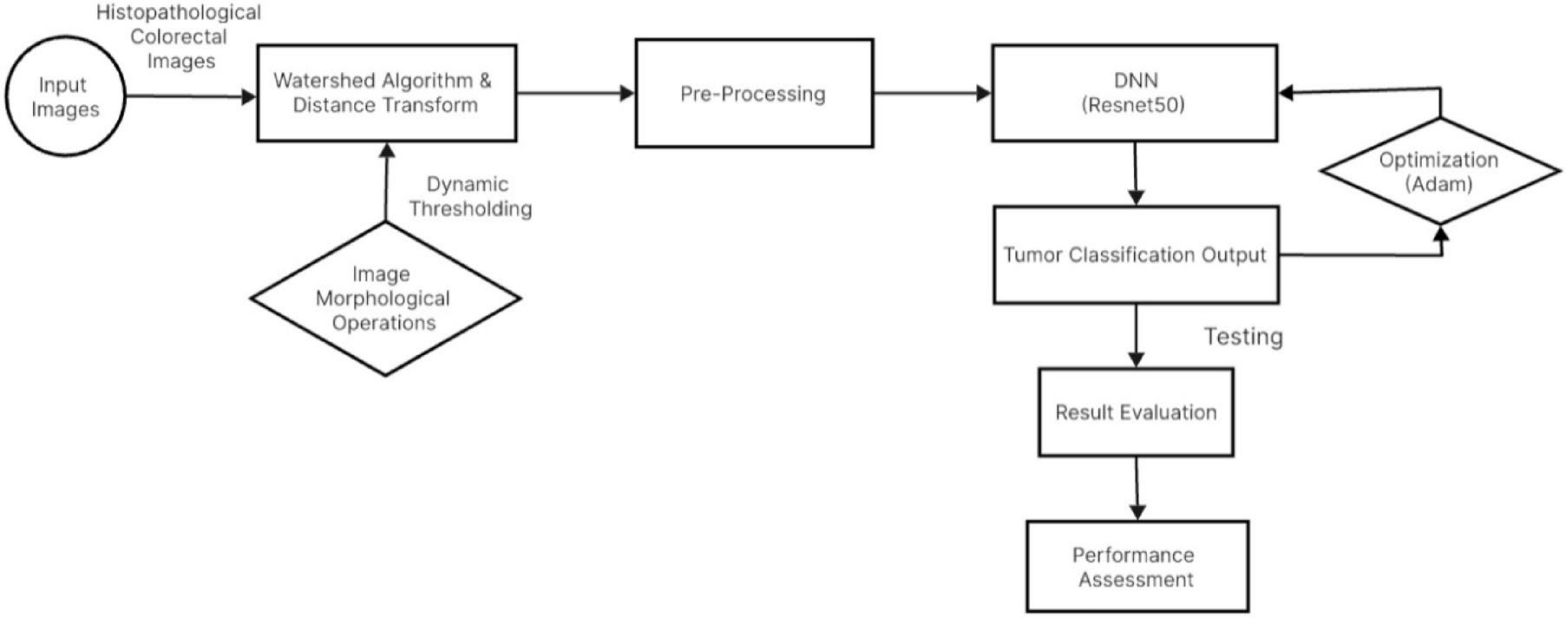
The overall framework of the proposed approach. It consists of image processing like Morphological Operations, Watershed Algorithm, and Distance Transform. Then DNN(Resnet50) is used with Adam for detecting the tumor images.

In order to improve the precision and interpretability of this research, hereby present a novel strategy in the work by integrating a double attention mechanism as in Fig. 4. The double attention mechanism functions in two stages: the first stage concentrates on identifying specific details in histopathological pictures that suggest colorectal tumors, and the second stage pays attention to important areas that the first stage of attention identified, enhancing the identification process. In addition to strengthening the model’s capacity to identify minute characteristics linked to malignant tissues, this dual attention approach reveals the precise regions that have the greatest influence on the ultimate diagnosis. The main goal is to improve the suggested diagnostic tool’s explainability and efficacy by including this twofold attention process, which will ultimately advance the field of medical image analysis for colorectal cancer detection.

The process begins with histopathological colorectal images containing different classes of tissue, including debris, adipose, complex structures, tumors, mucosa, stroma, lymph, and empty regions. These images serve as the starting point for tumor detection. The watershed algorithm and distance transform techniques are applied to the input images to segment them and identify potential tumor areas based on pixel distances. This step helps to separate different regions and isolate potential tumor regions from the surrounding tissue. Preprocessing involves applying vision transformation techniques to enhance image quality, remove noise, and standardize features. This ensures that the images are optimized for further analysis and classification, improving the accuracy of tumor detection. Convolutional neural network ResNet50 employs deep learning to distinguish between tumor and non-tumor zones in images and extract features that help identify tumors. The Adam optimizer is employed to optimize the performance of the ensemble deep neural network during training. It adjusts the network’s parameters iteratively to minimize loss and improve classification accuracy, ensuring better tumor detection results. The output of the ensemble deep neural network is a classification of the input images into tumor and non-tumor regions. This output provides valuable information about the presence and location of tumors within the colorectal images. The performance of the tumor detection system is evaluated using various metrics, including confusion matrix, accuracy, recall, precision, and the kappa measure (*k*). These metrics help assess the reliability and effectiveness of the detection algorithm, ensuring its suitability for clinical applications.

To mitigate class imbalance across the eight tissue categories (tumor, stroma, mucosa, debris, adipose, lymph, complex, empty), weighted loss functions and distributed class weights were employed. Additionally, Receiver Operating Characteristic (ROC) and Precision–Recall (PR) curves were analyzed for underrepresented classes, providing a more detailed evaluation of minority class performance.

## Architecture

Convolution layers are the major building blocks in image classifiers. The mathematical term convolution refers to the combination of two functions (f and g) that produce a third function (z). The convolutional layer takes an input, applies a filter, and outputs a feature map. The feature map (z) is a combination of input and filter (f and g), hence the name convolution layer. The objective of convolution is to extract the features of an image. A feature is a specific characteristic of the original image, such as points, edges, or shapes. Like the image being processed as numeric, a feature translates into a box of numeric pixel values. This matrix serves as a feature detector. To scan the image, this filter matrix moves across the image, pixel block by pixel block. For each subregion, a value is calculated based on how good of a fit there is between filter and image. The calculation is a simple multiplication of the two matrices. Therefore, the better the match the higher the resulting value in the feature map.

CNNs process the input data through a network of interconnected layers. Convolutional layers are often the first hidden layer in a CNN. They work by applying a series of filters to the input data in order to identify particular patterns. By swiping over the input data and multiplying each filter entry element-by-element, a feature map is produced for each filter. The model is then given non-linearities to learn more intricate patterns in the data by combining these feature maps and passing them via non-linear activation functions like the ReLU function.

**Fig. 5:**
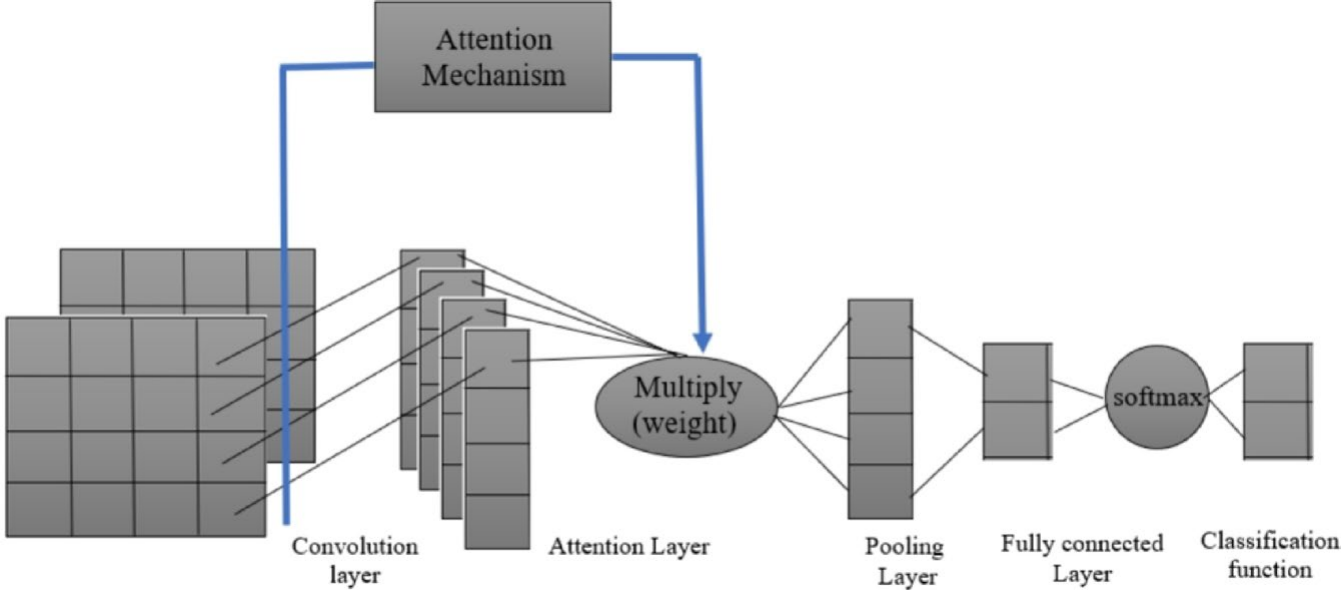
CNN Architecture used in the proposed System.

Additional convolutional layers, pooling layers, and fully-connected layers are examples of subsequent layers in a CNN. Feature maps are smaller when pooling layers are used. This improves the model’s computational efficiency by lowering the total number of parameters. In most CNNs, fully-connected layers may be found following the convolutional and pooling layers. The model may learn potential non-linear combinations of the features learnt by the convolutional layers thanks to fully-connected layers, which link all of the neurons in one layer to all of the neurons in the layer above. A CNN’s softmax layer, which generates a probability distribution over all potential class labels for the input data, is usually its last layer. The class with the highest probability is selected as the model’s forecast. To further improve its performance in image classification applications, CNN design uses regularization techniques and a double attention layer in addition to convolutional layers. Specifically positioned after the convolutional layers, the double attention layer enhances the model’s capacity to concentrate on important areas inside the feature maps. This layer is composed of two attention processes that work together: channel attention, which draws attention to significant feature channels, and spatial attention, which emphasizes pertinent spatial positions in the feature maps. The model’s ability to integrate these attention mechanisms allows it to reduce noise and prioritize important information, resulting in classification results that are more reliable and accurate (Fig. 5).

## Implementation

The training of the ensemble DNN model was divided into two major phases. The first phase training of individual architectures involves models, such as Inception-ResNet V2, and a custom CNN model architecture. Concatenating the outcomes from each individual model and building a deep ensemble network constitute the second phase. In addition, we use regularization strategies like dropout and L2 regularization to enhance generalization and avoid overfitting. A part of the neurons is randomly deactivated during training by dropout, which forces the model to rely on alternate paths and reduces the likelihood that it will memorize noise in the training set. Conversely, L2 regularization discourages the use of large model weights, promoting smoother decision limits and avoiding overcomplication. Figure 1 elaborates on the workflow. The configuration of the PC utilized for model creation was Intel^®^ Core i3-1115G4 @ 3.00 GHz 2.90 GHz. It is an innovative GPU with a higher 3500 CPU benchmark score. The multi-socket high-performance server microprocessor helped in providing faster computation during model evaluation and hence provided enhanced scope encompassing multiple.

### Dataset & preprocessing

**Fig. 6:**
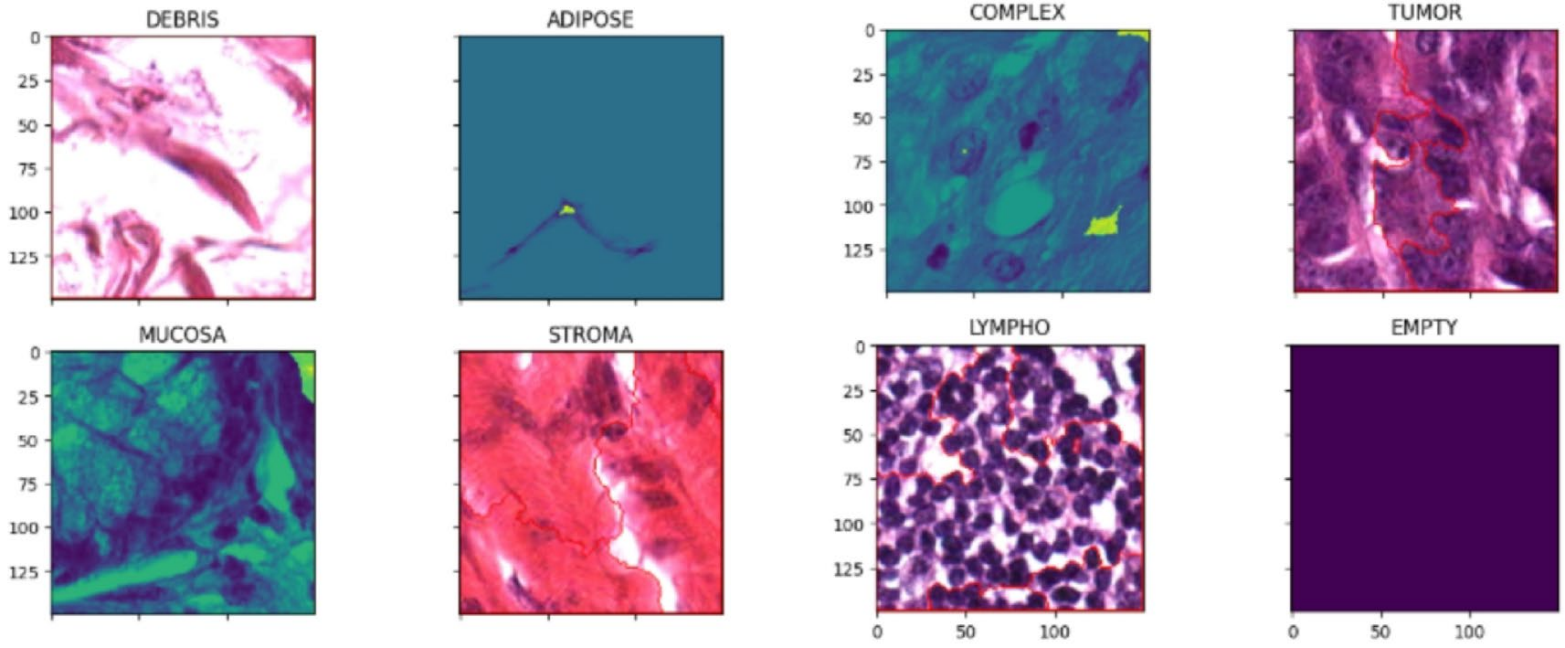
Types of Tissues used to Train the model (Tumour class & 7 benign classes).

The represent characteristics that are related to human cancer (CRC). The samples are collected from the accessible Histology (5000 images): The Kather et al., (2019) which has been created and open-sourced by and contains two zipped files. One of them consists of 5000 image tiles of size 150 × 150 pixels each resized to 100 × 100 and equally distributed into eight non-overlapping classes, namely STROMA, DEBRIS, ADIPOSE, MUCOSA, EMPTY, TUMOR, LYMPHO, and COMPLEX (625 tiles each). The other file consists of ten additional multi-class data files, each of size 5000 × 5000 pixels with multi-class tissue characteristics. The colour channels in the images are in RGB channel format, with 20× magnification (Fig. 6).

**Fig. 7:**
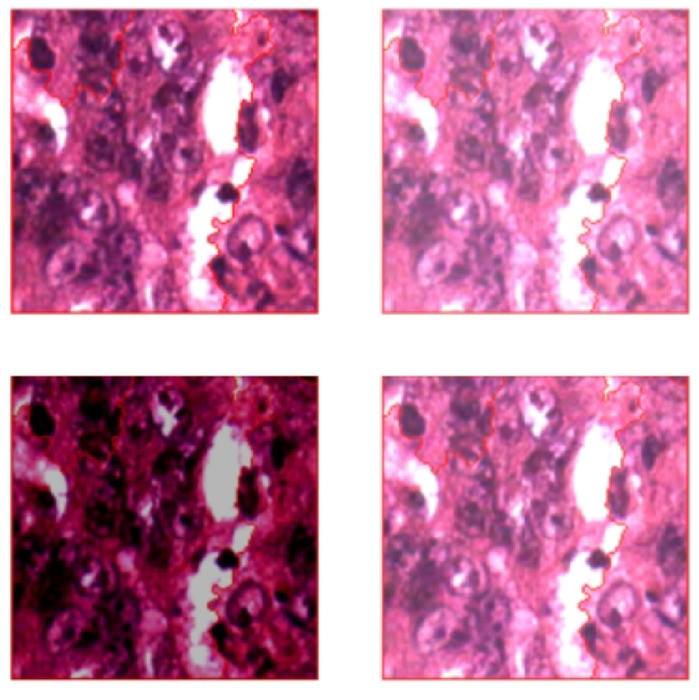
Tissue after boundary detection & augmentation.

This paper’s main purpose is to achieve maximum accuracy with less data compared to previous works. Scaling the features to a similar range is done using normalization and standardization techniques like log scaling & Z-Score. The images are augmented by changing the contrast, brightness & saturation and by adding rotations and flips. Contrast adjustment remaps image intensity using min-max adjustment which improves the clarity of the images used in medical analysis. In this paper there are several image processing techniques to enhance the analysis of colorectal histopathological images. Initially, Gaussian blur is applied to mitigate noise and improve segmentation accuracy. Subsequently, morphological operations are performed to refine the image and remove unwanted artifacts. Additionally, the distance transform method is utilized to identify potential tumour regions, aiding in the localization of abnormalities (Fig. 7; Table 1).

**Table 1:** Tissue Class, original Image, watershed Algorithm, distance transform: A comprehensive overview of image processing stages, from Raw data to segmented tissue classes, employing watershed algorithm and distance transform techniques.

Class	Original Image	Watershed Algorithm	Distance Transform
Tumour			
Stroma			
Complex			
Lymph			
Debris			
Mucosa			
Adipose			
Empty			

Furthermore, the watershed algorithm is integrated into the pipeline, which proved instrumental in segmenting intricate structures within the histopathological images. This comprehensive approach not only facilitated the segmentation of complex features but also enabled quantitative analysis essential for medical research and diagnosis. After applying these techniques to the dataset, the number of images is doubled. The enhanced images are then fed into the model for further analysis.

## Results & discussion

**Fig. 8:**
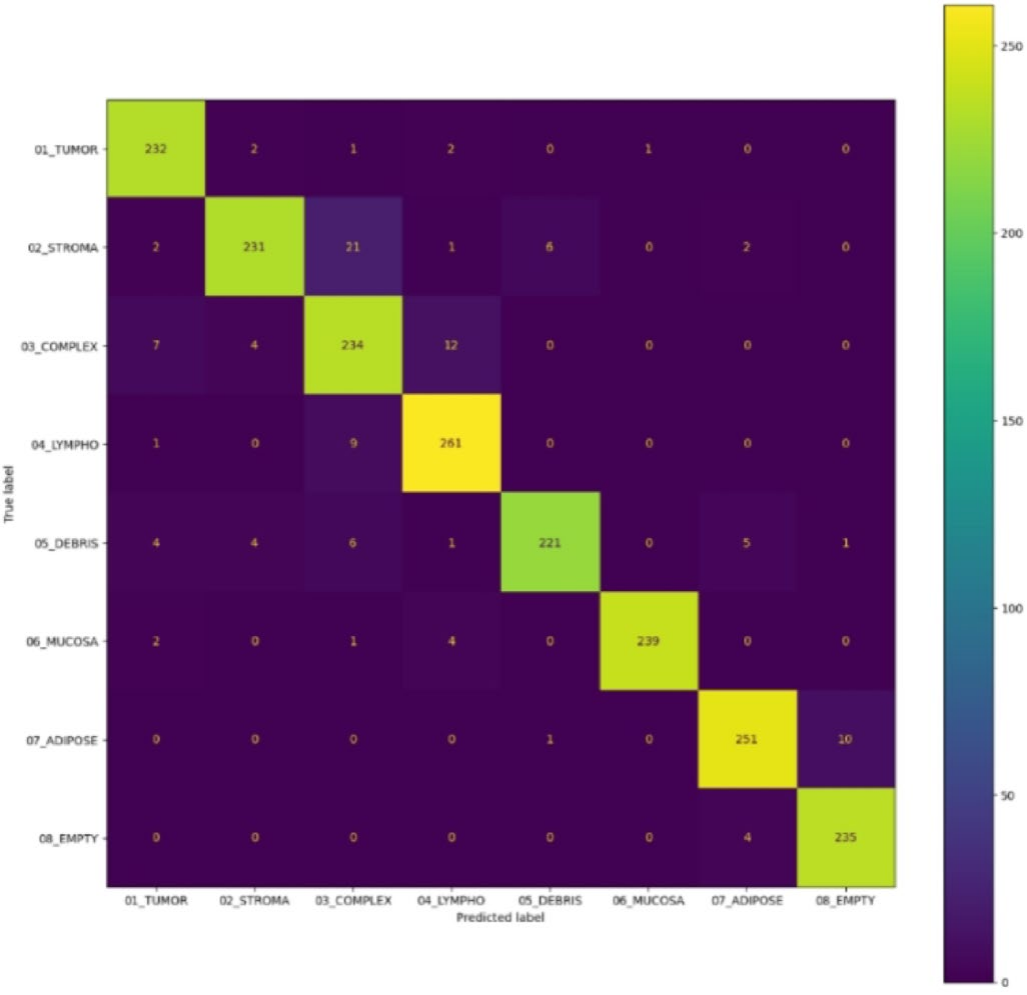
The resulting confusion matrix aligns true class labels along the x-axis and k-nearest neighbour’s class predictions along the y-axis. Correct classifications appear along the main diagonal, while misclassifications are evident elsewhere.

The proposed ensemble model was evaluated on the Kather et al. (2019) dataset of 5,000 colorectal histology images distributed across eight classes. The model achieved a training accuracy of 98.74%, validation accuracy of 94.35%, F1-score of 0.94, precision of 0.95, recall of 0.94, specificity of 0.96, and a Cohen’s kappa score of 0.9354, reflecting excellent inter-class agreement. Table 1 summarizes the class-wise performance metrics, including precision, recall, and F1-score for each tissue category based on formulae on Table 2. The confusion matrix (Fig. 8) highlights strong classification across most classes, with most predictions lying along the main diagonal. Misclassifications were primarily observed between tumor and stroma and complex and lymph nodes, which can be attributed to overlapping morphological features in histopathology. This demonstrates the challenge of tumor heterogeneity, a known limitation in colorectal cancer diagnosis.

**Table 2:** Formulas of the performance metrics used; key: true positives (TP), true negatives (TN), false positives (FP), and false negatives (FN) of the model’s predictions.

Accuracy	$\:\frac{TP+TN}{TP+TN+FP+FN}$
Precision	$\:\frac{TP}{TP+FP}$
F1 Score	2 × (Precision × Recall) / (Precision + Recall)
Recall	$\:\frac{TP}{TP+FN}$
Cohen’s Kappa (κ)	(Po − Pe) / (1 − Pe), where *Po* is observed agreement and *Pe* is chance agreement.

The proposed model has a kappa score of 0.9354 which tells us the excellent agreement with each appraiser and the higher the magnitude, the stronger the association. The kappa measure (*k*) measures the degree of agreement between a pair of variables, frequently used as a metric of interrater agreement. Generally, a kappa of less than 0.4 is considered poor (a Kappa of 0 means there is no difference between the observers and chance alone). Kappa values of 0.4 to 0.75 are considered moderate to good and a kappa of > 0.75 represents excellent agreement. Cohen’s kappa statistic, (*k*), is a proportion of arrangement between categorical variables (*ai*, …, *an*), where *n* is the total number of classes.

**Fig. 9:**
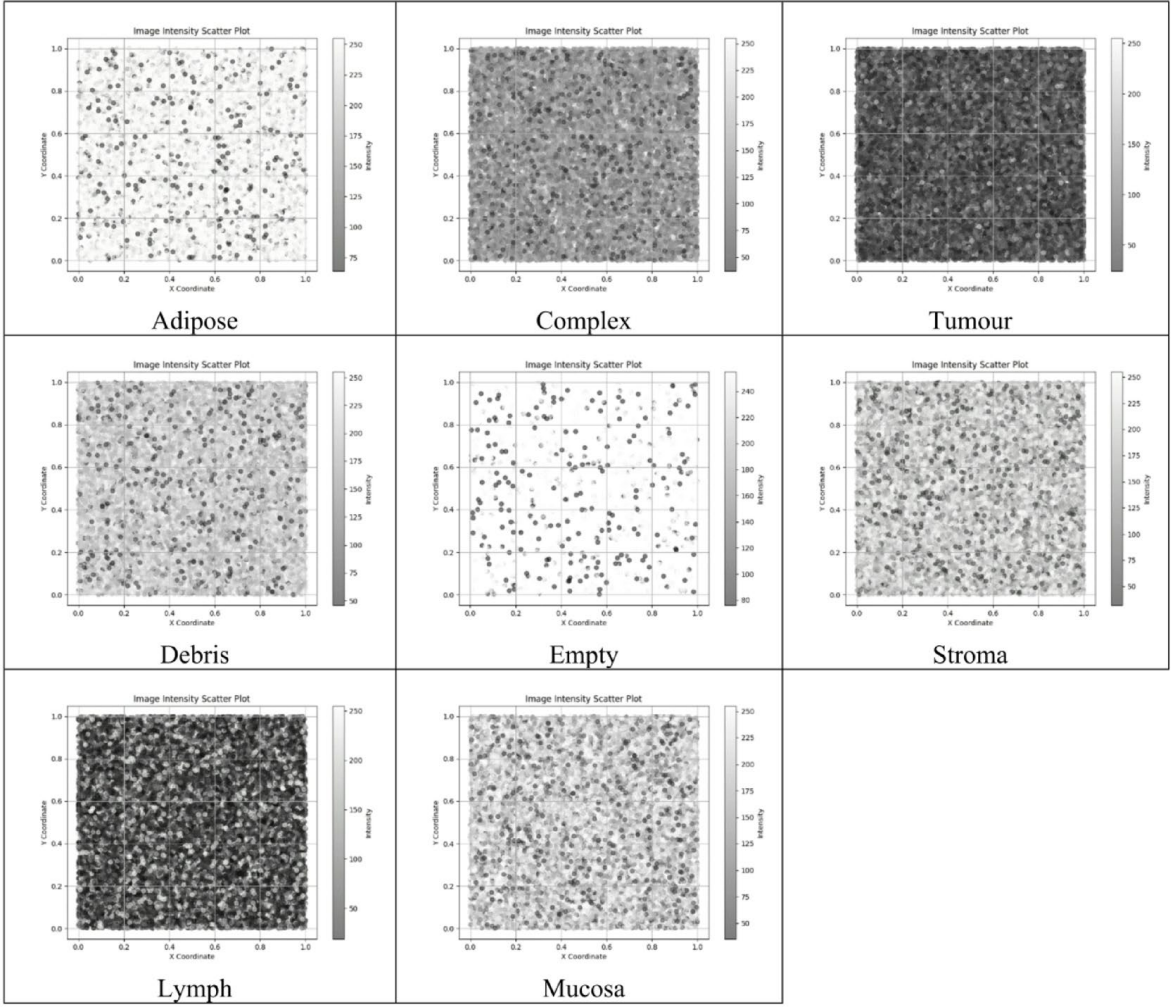
Scatter plot with intensity values of various classes in the dataset.

**Fig. 10:**
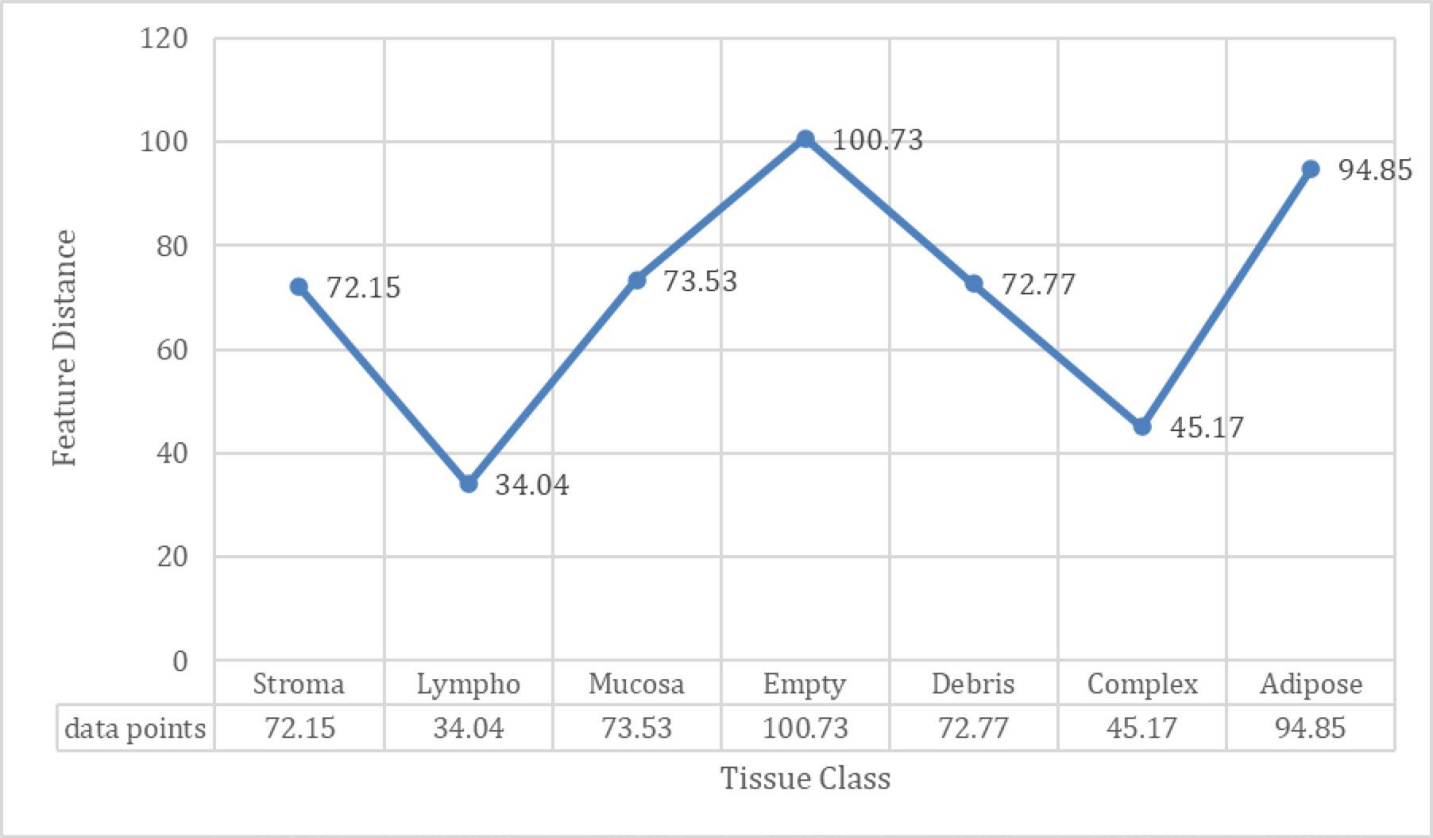
Feature Distance Comparison between Tumour and Benign classes (x-axis: different tissue classes; y-axis: obtained difference value of the feature distances).

**Fig. 11:**
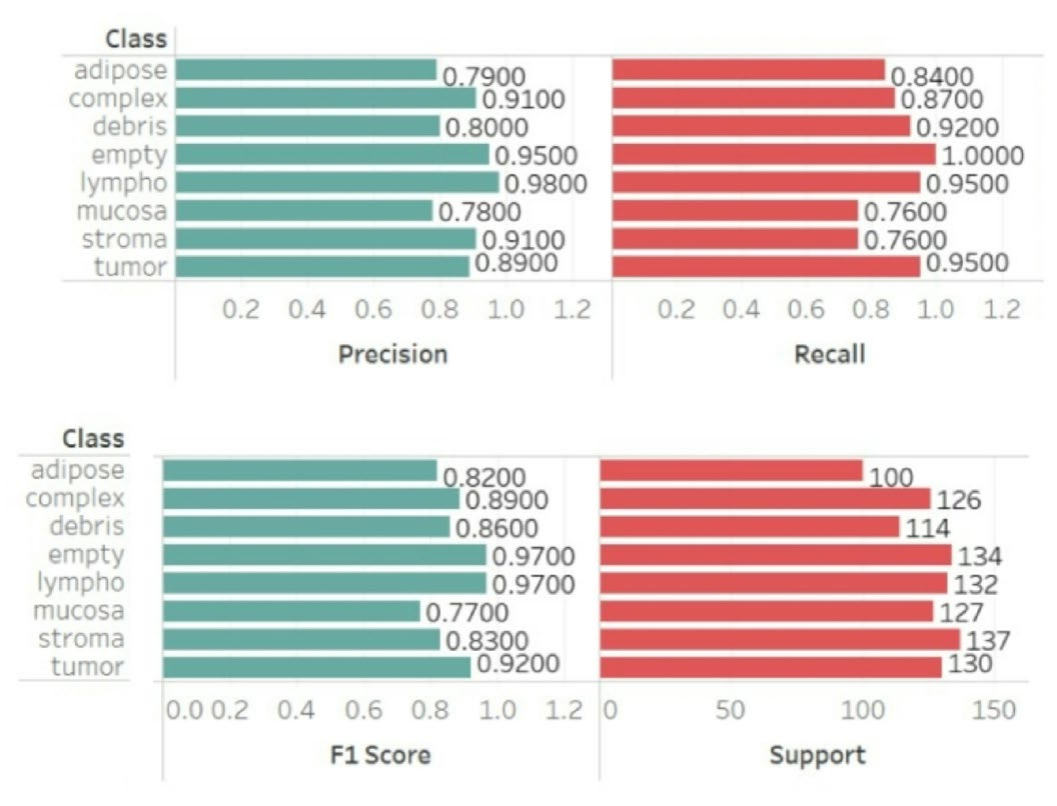
Class wise performance evaluation on Colorectal Histology dataset (x-axis: distinctive values of the parameters; y-axis: difference classes).

The line graph visualizes the feature distance comparison (Fig. 9) between scatter plot of tumour class and scatter plot across different benign tissue classes. Each tissue class, represented along the x-axis, exhibits a distinct difference between the distances of features from scatter plot 1 to scatter plot 2, depicted along the y-axis. Notably, the distances vary across tissue types, highlighting potential dissimilarities or similarities between the two scatter plots (Fig. 10). For instance, “empty” class has the largest difference of 100.73, indicating areas with no tissue or minimal cellular activity. “adipose” follows closely with a difference of 94.85, highlighting the unique intensity patterns of fat cells compared to tumours. “Mucosa” and “Stroma” show significant differences of 73.53 and 72.15, respectively, showing distinct intensity profiles associated with mucosal tissues and connective/supporting elements. “Debris” shows a similar difference of 72.77, probably representing scattered cellular remnants or non-cellular material. “Lymph” and “complex” show relatively smaller differences of 34.04 and 45.17, respectively, suggesting more subtle variations in intensity patterns associated with lymphocytes and complex tissue structures (Fig. 9). These intensity differences highlight the distinct histological composition and structural characteristics of each class relative to tumour tissue, aiding in accurate classification and histopathological analysis. Through this visual representation, the line graph provides insight into the comparative structural relationships between different tissue classes (Fig. 11), offering a comprehensive understanding of their overall spatial distribution and arrangement.

**Fig. 12:**
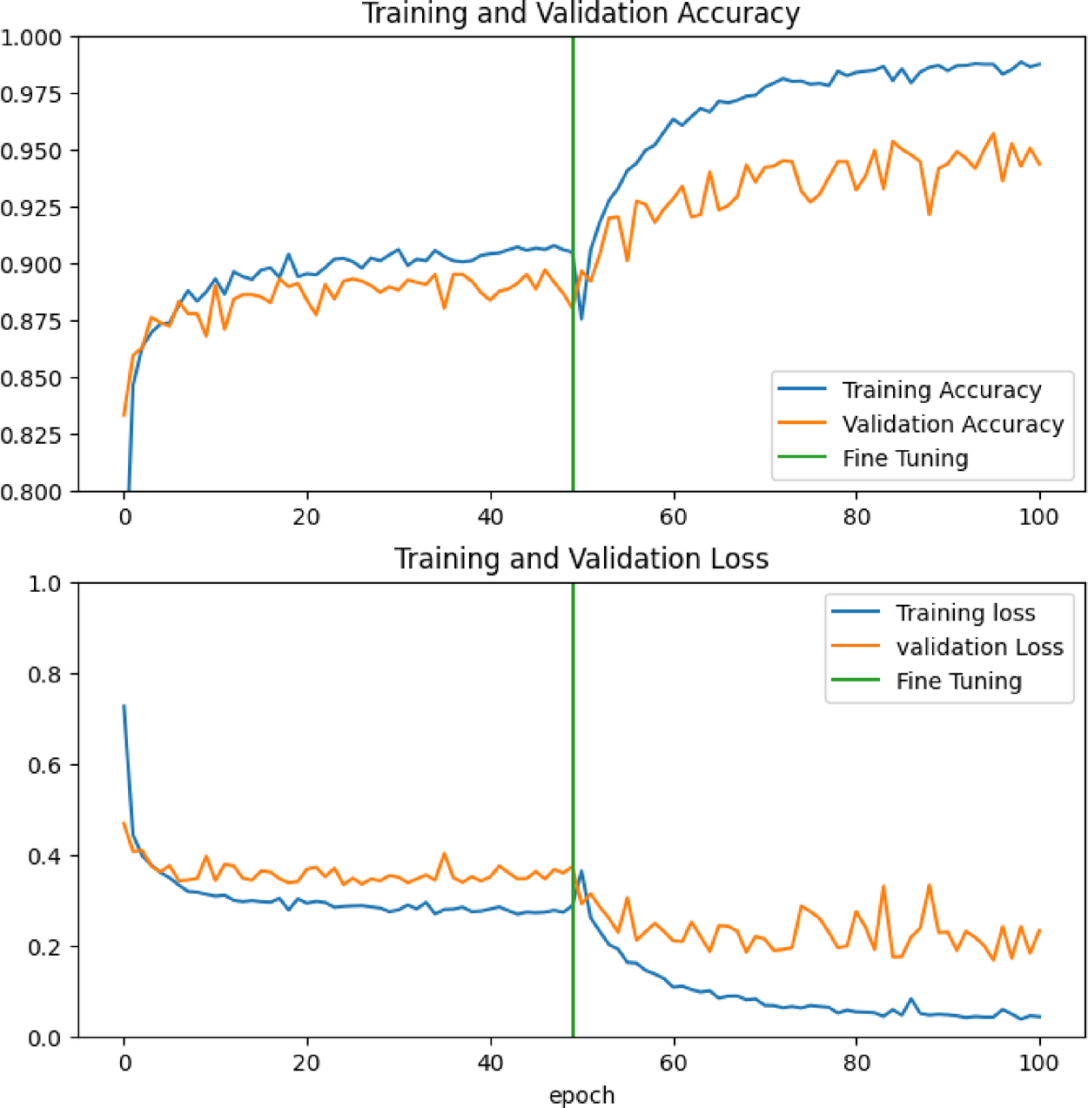
Results of Training and validation Accuracy & Loss (x-axis: no. of Epochs; y-axis: obtained value at each epoch).

This work has made significant improvements to the field of medical image analysis, particularly when it comes to the application of deep learning methods for the diagnosis of colorectal tumours. Although earlier efforts in this area produced impressive outcomes using a bigger dataset of 10,000 photos, this method concentrated on a more restricted collection of 5,000 images. Even with this decrease in the amount of data, model performed well, with an accuracy rate of 94% (Fig. 12). Interestingly, this result was obtained with fewer training epochs, demonstrating the validity and efficiency of the suggested methodology. Significant regions within the histopathology pictures were highlighted and interpretability was greatly improved by the addition of attention mechanisms. This helps to better understand the model’s decision-making process and offers useful information to medical professionals on the location and identification of possible colorectal cancers (Fig. 12).

It is important to note that the model was trained over 100 epochs at a fixed learning rate. This intentional decision was made with the goal of maintaining a stable and broadly applicable performance by finding a balance between preventing overfitting and promoting model convergence. The choice to utilize a constant learning rate highlights the durability of the methodology and its flexibility in different training scenarios.

### Limitations

The absence of external validation and the reliance on a single dataset may restrict generalizability. Differences in staining protocols or clinical imaging conditions could impact real-world applicability.

### Future work

To enhance robustness, future studies could incorporate multi-center datasets, explore multi-modal fusion (e.g., radiology + histopathology), and investigate real-time deployment strategies for integration into clinical workflows^39^.

Overall, these results establish the proposed ensemble with double attention as a reliable and interpretable framework for colorectal tumor detection, advancing the potential for AI-assisted pathology.

## Data Availability

The data that support the findings of this study are openly available at [https://zenodo.org/record/53169#.W6HwwP4zbOQ](https:/zenodo.org/record/53169) with DOI 10.5281/zenodo.53169 Kather JN, Weis CA, Bianconi F, Melchers SM, Schad LR, Gaiser T, Marx A, Zollner F: Multi-class texture analysis in colorectal cancer histology (2016), Scientific Reports.
